# A systematic review on pharmacokinetics, cardiovascular outcomes and safety profiles of statins in cirrhosis

**DOI:** 10.1186/s12876-021-01704-w

**Published:** 2021-03-16

**Authors:** Shuen Sung, Mustafa Al-Karaghouli, Sylvia Kalainy, Lourdes Cabrera Garcia, Juan G. Abraldes

**Affiliations:** 1 Faculty of Medicine and Dentistry, University of Alberta, Edmonton, AB Canada; 2 Alberta Health Services, Edmonton, AB Canada; 3 Faculty of Medicine, Complutense University of Madrid, Madrid, Spain; 4 Division of Gastroenterology, 1-38 Zeidler Ledcor Centre, University of Alberta, 8540 112 St NW, Edmonton, AB T6G 2X8 Canada

**Keywords:** Liver, Hepatic, Rhabdomyolysis, Portal hypertension, Myalgia, Drug induced liver injury

## Abstract

**Background/Aims:**

There is increased interest in the therapeutic use of statins in cirrhosis, but preferred statin and safety outcomes are still not well known. In this systematic review we aimed to address pharmacokinetics (PK), safety, and effects on cardiovascular (CV) outcomes of statins in cirrhosis.

**Methods:**

Our systematic search in several electronic databases and repositories of two regulatory bodies up to 2020-06-11 yielded 22 articles and 2 drug monographs with relevant data.

**Results:**

Rosuvastatin and pitavastatin showed minimal PK changes in Child–Pugh A cirrhosis. Only rosuvastatin was assessed in a repeated dosing PK study. Atorvastatin showed pronounced PK changes in cirrhosis. No PK data was found for simvastatin, the most commonly used statin in cirrhosis trials. There was insufficient data to assess CV effects of statins in cirrhosis. Clinical trials in cirrhosis were limited to simvastatin, atorvastatin, and pravastatin. In patients taking simvastatin 40 mg, pooled frequency of rhabdomyolysis was 2%, an incidence 40-fold higher than that reported in non-cirrhosis patients, while this was no rhabdomyolysis observed in patients on simvastatin 20 mg, atorvastatin 20 mg, or pravastatin 40 mg. Drug-induced liver injury was of difficult interpretation due to co-existence of muscle damage. No overt liver failure was reported.

**Conclusions:**

Simvastatin 40 mg should be avoided in decompensated cirrhosis. Safety data on simvastatin 20 mg or other statins are based on small study sample size. This rarity of evidence combined with lack of data in dose adjustment methods in cirrhosis is a barrier for using statins for CV indications or for investigational use for liver indications.

**Supplementary information:**

The online version contains supplementary material available at 10.1186/s12876-021-01704-w.

## Background

Hepatic drug clearance is a complex process that is dependent on hepatic blood flow, intrinsic hepatic clearance and fraction of unbound drugs in blood [1, 2]. Within the liver, phase I and II metabolism are responsible for the processing of different medications. Furthermore, biliary excretion is an important means for drug elimination [1]. In cirrhosis, many of these processes may be affected which, in turn, will impact the overall metabolism and clearance of medications. In addition, the presence of portal systemic shunting, concurrent renal impairment and hepatic toxicity of certain medications could alter their effectiveness and adverse effects. Therefore, in patients with cirrhosis, assessment of liver function and portal-systemic shunt would be ideal to properly adjust drug doses. However, due to the complexity of hepatic drug elimination, it is difficult to develop a universal method of dose adjustment [1].

Child–Pugh classification has been used to assess prognosis of chronic liver disease, primarily in cirrhosis. The Food and Drug Administration (FDA) and European Medicines Agency (EMA) recommend to characterize the pharmacokinetics of medications that undergo extensive hepatic metabolism or with narrow therapeutic index during drug development [3, 4]. The Model for End-stage Liver Disease (MELD) score is used to predict survival in cirrhosis and reflects the severity of liver and kidney dysfunction [5, 6]. Despite their roles in liver function evaluation, both scoring systems may not accurately reflect the liver’s ability to metabolize medications [1]. Furthermore, it remains unclear if the application of these methods to adjust medication doses would have an impact on efficacy and safety outcomes of various hepatically metabolized medications.

Hydroxymethylglutaryl-CoA (HMG-CoA) reductase inhibitors, commonly known as statins have demonstrated significant benefits on cardiovascular (CV) mortality and morbidity in the general population [7]. Recently, more attention has been focused on statins’ potential hepatic benefits and their use in patients with cirrhosis [8] , with an increasing number of studies showing improvement in portal hypertension and clinical outcomes. These have been extensively summarized in recent systematic reviews [9–11]. Nonetheless, currently the only indication of statins in cirrhosis remains cardiovascular disease prevention [8].

There is still uncertainty on whether the cardiovascular benefits of statins apply to patients with cirrhosis as this population was excluded from major statin trials [12–14] , likely due to predicted higher rate of adverse effects such as myopathy and potential liver toxicity [15]. This, together with the paucity of data on safety of statins in cirrhosis, makes it difficult to assess the balance of benefits and risks of statins in cirrhosis. This knowledge will be especially important in a context of increasing proportion of patients with cirrhosis with a non-alcoholic fatty liver disease etiology, who have a high cardiovascular risk [16]. Another barrier for the use of statins in cirrhosis is the lack of a validated hepatic dose-adjustment method, which requires a thorough understanding of the pharmacokinetics of statins in cirrhosis. Solute carrier (SLC) membrane transporters, such as organic anion transporting polypeptide (OATP) 1B1, are found in the liver and are responsible for the transport of multiple statins from the portal vein into the hepatocytes [17–19]. Many statins undergo metabolism by cytochrome P450 (CYP450) enzymes in the hepatocytes and are eliminated by biliary excretion [17, 20]. The process of biliary excretion is carried out by ATP-binding cassette (ABC) transporters such as multidrug resistance protein 1 (MDR1) as known as P-glycoprotein [17]. Cirrhosis has been associated with abnormalities on the expression of SLC membrane transporters, ABC transporters and CYP enzymes [21–23]. Hence, substantial pharmacokinetic changes of statins in cirrhosis can be expected.

Therefore, this research project had three objectives. First, we aimed to systematically search for available evidence informing pharmacokinetic changes of statins in cirrhosis (research question one). Second, we aimed at identifying the impact of statins on cardiovascular outcomes and safety profiles specifically in patients with cirrhosis (research question two). Lastly, we investigated if there is evidence to support any of the several potential hepatic dose adjustment methods (Child–Pugh scores, MELD score or clinical gestalt) when prescribing statins to patients with cirrhosis (research question three).

## Methods

### Search strategies

This systematic review searched for available evidence pertaining to the three research questions in the pre-specified databases. The following databases were searched for available data up to June 11, 2020: MEDLINE via Ovid, EMBASE, Cochrane Library, CINAHL, and SCOPUS. Since all three research questions were related to patients with cirrhosis taking statin medications, a single search strategy was developed to capture relevant research. In consultation with a librarian at the University of Alberta, the initial search strategy was first developed and later modified in the MEDLINE database via Ovid. In MEDLINE, the final search strategy included subject headings “liver cirrhosis” or “liver cirrhosis, alcoholic” or “liver cirrhosis, biliary”, and searched title, abstracts, and keywords using terms “"Liver cirrhosis" or "cirrhosis" or "liver fibrosis" or "liver failure" or "alcohol liver cirrhosis" or "biliary cirrhosis" or "compensated liver cirrhosis" or "decompensated liver cirrhosis" or "primary biliary cirrhosis". The results from searches in subject headings and title, abstracts and keywords were combined using the “OR” function to generate a collection of articles related to cirrhosis (this result will be referred to as collection 1 from here on). Statins were searched with subject headings “hydroxymethylglutaryl-coa reductase inhibitors” or “atorvastatin” or “lovastatin” or “pravastatin” or “rosuvastatin calcium” or “simvastatin” and with the following title, abstracts, and keywords “statin* or atorvastatin* or lovastatin* or pravastatin* or rosuvastatin* or simvastatin* or fluvastatin* or lipitor or crestor or lescol or zocor or pravachol or mevacor or HMG-CoA reductase inhibitor* or pitavastatin* or livalo or hydroxymethylglutaryl-coa reductase inhibitor*”. The results from searches in subject headings and title, abstracts and keywords were combined using the “OR” function to generate a collection of articles related to cirrhosis (this result will be referred to as collection 2 from here on). Collection 1 and collection 2 were combined using the “AND” function to generate a collection of articles related to both cirrhosis and statins. Note that pitavastatin was not available as subject headings in MEDLINE via Ovid and therefore was not among the subject headings above. Using these search terms, searches were completed similarly in the other databases stated above. Please see Additional file 1: supplementary data for full description of search strategy used in each database.

In addition to the above search strategies, Health Canada Drug Product Database (https://www.canada.ca/en/health-canada/services/drugs-health-products/drug-products/drug-product-database.html) and Drugs@FDA database (https://www.accessdata.fda.gov/scripts/cder/daf/) were searched for drug product monographs, labels, medical and/or pharmacology reviews pertaining to pharmacokinetics of simvastatin, pravastatin, rosuvastatin, atorvastatin, fluvastatin, lovastatin, and pitavastatin. Search terms were the generic names of the statins, and documents were reviewed and retrieved in the respective brand name products.

### Study selection

Inclusion criteria were slightly different for each of the three research questions. The shared inclusion criteria for study types were randomized controlled trials, cohort studies, case-controlled studies, and case series. Pharmacokinetic studies were included for research question one. All studies included were published in English language and involved patients over age of 18 years old, with diagnosis of cirrhosis, any indications for statins and taking any statins.

Outcomes of interest for research question one were changes in absorption, distribution, protein binding, biliary excretion, metabolism, excretion, and renal elimination. For research questions two and three, outcomes of interest were divided into efficacy and safety outcomes. For research question two, efficacy outcomes included cardiovascular mortality, non-fatal myocardial infarction, non-fatal stroke, major cardiovascular adverse events, and thrombotic events. For research question three, efficacy outcomes included all-cause mortality, cardiovascular mortality, non-fatal myocardial infarction, non-fatal stroke, major cardiovascular adverse events, thrombotic events, hospitalization rate due to any cause, complications of cirrhosis, and mortality due to cirrhosis. Safety outcomes were the same for both research questions two and three, which included muscle injury, rhabdomyolysis, deterioration of liver function tests, deterioration of existing liver disease, gastrointestinal adverse effects, hemorrhagic stroke, diabetes mellitus, and cognitive impairment. Exclusion criteria included animal studies and case reports.

**Fig. 1:**
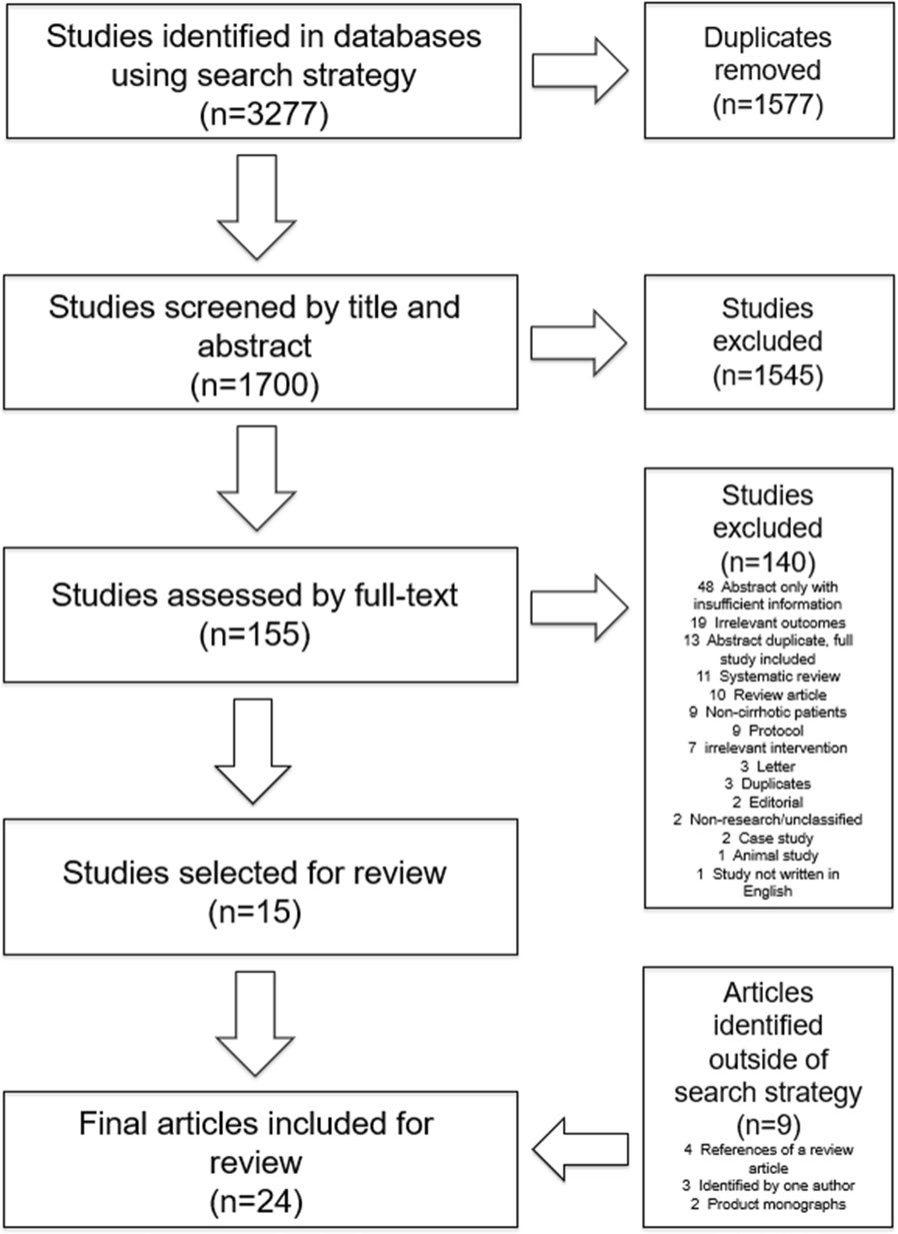
Study selection process: Overall, a total of 3277 articles were identified using the above mentioned search strategy from the pre-specified databases. 1577 studies were removed as duplicates and the remaining 1700 studies were screened using the pre-specified inclusion and exclusion criteria. After title and abstract screening, 1545 studies were deemed irrelevant, and the remaining 155 articles were reviewed by full-text. 15 studies were selected for inclusion of the systematic review. Additional 9 articles/product monographs were identified outside of the search strategy and included for the review

Study selection process was carried out using Covidence web-based tool (description of Covidence can be found on https://www.covidence.org/). Articles were imported to Covidence online tool after initial search was completed. Two independent reviewers (SS and SK) screened all articles by title and abstract using the inclusion/exclusion criteria specified above. Discrepancies in decision were resolved by a third reviewer (JGA). Full text reviews were completed by two independent reviewers (SS and JGA). Discrepancies were resolved by consensus. Summary of the study selection process can be found in Fig. 1.

Articles and documents from Health Canada Drug Product Database and Drugs@FDA database were selected if information on pharmacokinetics in cirrhosis was identified. This process was completed by one reviewer (SS) and was not part of the systematic search strategy.

Systematic reviews were only included for review of their bibliography to extract additional relevant articles; the systematic reviews selected during abstract and full text screening were not included for data analysis. Additional articles selected from the bibliography of systematic reviews were reviewed by two independent reviewers (SS and JGA) to determine if eligible for inclusion.

### Data extraction

Data extraction was performed by two independent reviewers (SS and MA). Discrepancies in decision were reviewed by reviewers and consensus was reached either by discussion or decision by a third reviewer (JGA). Data extraction was carried out using Microsoft®Excel® spreadsheet. The following data were extracted: authors/publication year, study type, number of participants, duration of study, participant characteristics, relevant inclusion/exclusion criteria, intervention, efficacy outcomes, safety outcomes, and pharmacokinetic outcomes.

### Quality assessment

Quality assessment of selected studies was performed by two independent reviewers (SS and MA). Randomized controlled trials were assessed using the Cochrane Risk of Bias Tool [24] and observational studies were assessed by Newcastle–Ottawa Quality Assessment Scale [25]. Discrepancies in decision were solved by consensus or decision by a third reviewer (JGA).

### Statistical analysis

Data extracted from articles was reported in mean values unless it was not specified in the original article. If mean values were not available but median values were available, estimation of mean values was done as previously described [26, 27]. Quantitative meta-analysis was performed only for the safety outcomes due to lack of data for other outcomes. For meta-analysis of proportions these were transformed using the double arcsine method and pooled with a random-effects model using the restricted maximum likelihood method. These analyses were performed in R using the *meta* package. Binomial probabilities reported in the discussion were estimated using an online calculator from University of Iowa [28].

## Results

Figure 1 shows the study selection process. Initial search identified 15 studies. Search in the references of published systematic reviews [9–11, 29, 30] , did not identify additional articles. Search in the reference list of one review article [8] identified four additional articles. One of the authors identified an additional article [31] that was published after initial search was completed. Two additional articles [32, 33] that were not indexed in any of the databases were identified outside of the search strategy and included for the review. Searches in Health Canada Drug Database and Drugs@FDA database identified two drug product monographs [34, 35] containing pharmacokinetics information. Therefore, 22 articles and 2 product monographs were included for this systematic review. Of these, 4 articles and 2 product monographs were pertinent to research question one and 18 articles were pertinent to research question two. The search did not identify any articles that pertained to research question three.

### Pharmacokinetics of statins in cirrhosis

Additional file 1: Table S1 summarizes available data on pharmacokinetics of statins in cirrhosis. Three were original studies [36–38] , with a total of 58 participants with Child–Pugh class A or B. Of these, two were single-dose studies [36, 37] with pitavastatin and fluvastatin. The third was a short-term study in which rosuvastatin was given for 14 days [38]. An additional review article [39] contained relevant information on pravastatin pharmacokinetics. Finally, drug product monographs [34, 35] for atorvastatin and pitavastatin reported pharmacokinetics information. Of note, none of these provided information related to Child–Pugh class C patients.

Overall, pharmacokinetic changes related to cirrhosis were identified for atorvastatin, fluvastatin, pitavastatin, pravastatin, and rosuvastatin (Additional file 1: Table S1). The largest change of area under the curve (AUC) and maximum plasma concentration (Cmax) was found in Child–Pugh class B patients taking atorvastatin, which was 11-fold and 16-fold increase respectively [34]. As this information was derived from drug product monograph, there was no information on baseline characteristics of the patients or dosing. The smallest change in AUC was reported in Child Pugh A participants compared to controls taking rosuvastatin 10 mg daily. [38] The smallest change in Cmax was reported in Child Pugh A participants compared to controls after a single dose of pitavastatin 2 mg [36]. In general, all available information showed higher AUC and *C*_max_ in Child–Pugh class B participants compared to Child–Pugh class A participants. Data in fluvastatin [37] and pravastatin [39] did not specify the Child–Pugh classes of participants. We did not find information on pharmacokinetic changes related to lovastatin and simvastatin in cirrhosis.

### Cardiovascular efficacy of statins in patients with cirrhosis

Only one retrospective cohort study [40] and one single arm trial [32] reported cardiovascular outcomes. Kaplan et al. showed a lower rate of major adverse cardiovascular events (MACE) in non-initiator (defined as patients who never started on statin therapy before and after diagnosis of cirrhosis) compared to existing user (defined as patients who were already on statin therapy before diagnosis of cirrhosis) with unadjusted hazard ratio (HR) of 0.58 [40]. The presence of likely unresolved residual confounding after adjusted analysis makes it difficult to draw conclusions on the efficacy of statins from this study. Munoz et al. reported no cardiovascular events and adverse events in participants taking simvastatin after a median (or mean) follow up of 87.0 ± 50.8 months [32].

### Safety of statins in patients with cirrhosis

The most common safety outcomes were rhabdomyolysis, muscle injury not meeting the criteria of rhabdomyolysis, myalgia, and drug-induced liver injury (AST/ALT increase). These are summarized in Table 1.

**Table 1:** Quantitative summary of the safety outcomes with different statins

Statin/dose	Authors/publication year	Composite of rhabdomyolysis and muscle injury	Rhabdomyolysis	Muscle injury not fulfilling criteria of Rhabdomyolysis	Myalgia	Drug induced liver injury
Simvastatin 40 mg	Abraldes 2009 [48]	2/30	0/30	2/30 ↑CK > 2x	Muscle weakness or myalgias 1/30	1/30 ↑AST > 2x
	Abraldes 2016 [51]	2/70	2/70 (one Child B the other in Child C)	0/70	0/70	1/70 ↑3 × liver transaminases
	Elwan 2018 [63]	0/20	0/20	0/20	2/20	0/20
	Pollo-Flores 2015 [49]	0/14	0/14	0/14	1/14	0/14 ALT decreased in simvastatin group, no values provided, and did not reach significance
	Pose 2019 [59]	3/16	3/16 (2 Child Pugh C, 1 Child Pugh B)	0/16 CK—mean difference vs. placebo: 1009 IU/L (208 to 1809) p = 0·014	(muscle symptoms) 5/16	3/16 ↑AST or ALT > 3 × ULN *AST—mean difference vs. placebo*: 130 IU/L (54 to 205) p = 0.0009 *ALT -mean difference vs. placebo:* 61 IU/L (22 to 100) p = 0·0025
	Vijayaraghavan 2020 [50]	3/81	*n* = 3/81 (all Child Pugh C)	0/81	0/81	*n* = 3/81 AST/ALT > 20 × ULN (same patients as Rhabdomyolysis)
	*Jha 2019 [33] (*n* = 65 in simvastatin group)	0/65	0/65	0/65	0/65	0/65
	*Munoz 2019 [32] (*n* = 9 in simvastatin group)	0/9	0/9	0/9	0/9	0/9
	Munoz 2018 [44] (abstract) (Munoz 2020 [45] full article published after initial search)	4/30	0/30	4/30 (13%) myalgia plus CK increase	7/30 (23%)	0/30
		Total: 14/335 (14/261 excluding*)	Total: 8/335 (8/261 excluding*)	Total: 6/335 (6/261 excluding*)	Total: 16/335 (16/261 excluding*)	Total: 8/335 (8/261 excluding*)
Simvastatin 20 mg	Pose 2019 [59]	N/A	0/14	0/14	(muscle symptoms) 6/14	1/14 ↑AST and ALT > 3 × ULN
	Wani 2017 [64]	N/A	0/38	1/38—muscle weakness, CPK > 5X ULN with normal ALT (after 15 days of simvastatin 20 mg daily before dose titration)	0/38	0/38
		N/A	Total: 0/52	Total: 1/52	Total: 6/52	Total: 1/52
Atorvastatin 20 mg	Bishnu 2018 [42] (*n* = 11 for atorvastatin group)	N/A	0/11	0/11	0/11	0/11
	Ghadir 2019 [53] (both intervention and control group has atorvastatin)	N/A	0/40	0/40	0/40	7/40 (3/20 + 4/20) ↑ liver transaminases > 3 xULN or noncompliance were excluded from final analysis
		N/A	Total: 0/51	Total: 0/51	Total: 0/51	Total: 7/51
Pravastatin 40 mg	Jouve 2019 [52] (84.8% of participants with cirrhosis) All patients received Sorafenib	N/A	0/155	CK increase Grade 1–2 29/155 in Sorafenib + Pravastatin **vs** *(30/157 in Sorafenib alone)* Grade 3–4 1/155 in Sorafenib + Pravastatin vs. 0/157 in sorafenib alone	Grade 1–2 24/155 in Sorafenib + Pravastatin vs *(19/157 in Sorafenib alone)* Grade 3–4 3/155 in Sorafenib + Pravastatin vs. 0/157 in sorafenib alone	0/155
	Riaño 2020 [31] (90.3% of participants with cirrhosis) All patients received Sorafenib	N/A	0/15	0/15	0/15	0/15
	Blanc 2018 [43] (Abstract)	N/A	N/A	N/A	N/A	N/A
		N/A	Total: 0/170	Total: 30/170	Total: 27/170	Total: 0/170

^*^Published in journals not indexed in the studied databases

#### Muscle safety

**Fig. 2:**
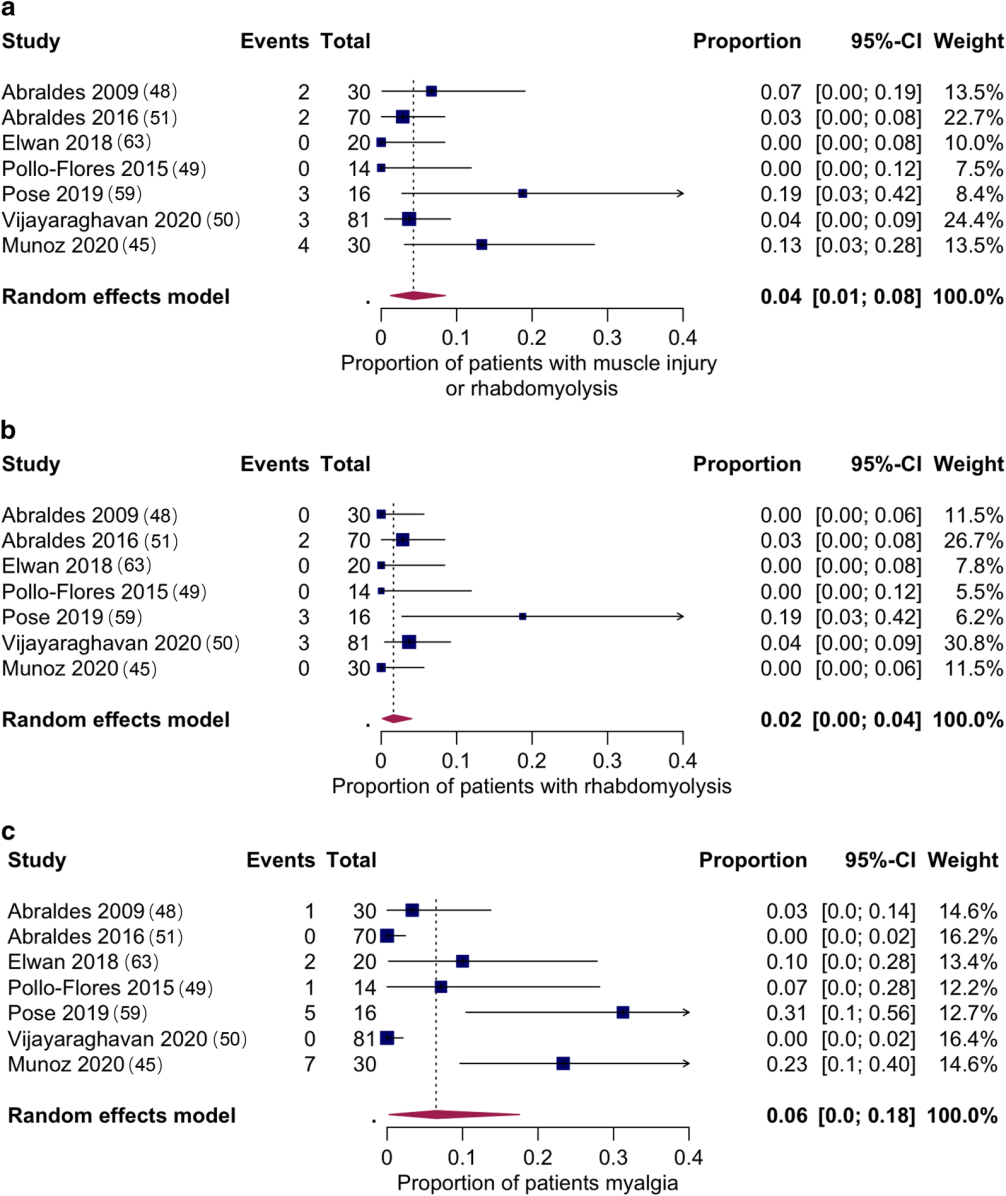
Pooled proportions of patients on simvastatin 40 mg daily that experienced muscle injury or rhabdomyolysis (**a**), rhabdomyolysis (**b**) and myalgia (**c**). Two studies [32, 33] were excluded from the quantitative meta-analyses since they were not indexed in any of the probed databases, had overall poor quality and had a high risk for bias

Quantitative meta-analyses was only possible for adverse effects of simvastatin 40 mg once daily. Figure 2 shows the pooled proportion of patients with rhabdomyolysis, any muscle injury (a composite of muscle injury or rhabdomyolysis) and myalgia. Two studies [32, 33] were excluded from the quantitative meta-analyses (poor overall quality, not indexed in any of the probed databases and high risk for bias). Point estimates for the proportion of patients that experienced events were 2% (95% CI 0–4%) for rhabdomyolysis. Of note, all instances of rhabdomyolysis reported with simvastatin 40 mg daily occurred in Child B and C patients, and none in Child A. There were no reports of rhabdomyolysis with simvastatin 20 mg, pravastatin 40 mg or atorvastatin 20 mg.

The pooled proportion of patients with muscle injury or rhabdomyolysis was 4% (95% CI 1–8%), and for myalgia 6% (95% CI 0–18%). Only myalgia showed significant heterogeneity.

#### Liver safety

Drug induced liver injury was reported in users of simvastatin 20 mg and 40 mg, and atorvastatin 20 mg, though this was of difficult interpretation due to the concomitant increase in ALT/AST induced by muscle injury. In a cohort study, Patel et al. studied the safety of statin in patients with decompensated cirrhosis waiting for liver transplantation [41]. Although the specific types of statins were not stated, the authors reported no significant increase of AST, ALT, bilirubin, and MELD score in the statin group, and the risk of hospitalization was similar between the statin group versus non-statin groups [41].

Lastly, one study involving atorvastatin users which lasted for one year did not report safety data [42].

#### Additional results from studies reported in abstract form (*n* = 3)

There was a total of 157 participants in a RCT and 1221 participants in two observational studies (Additional file 1: Table S2). Blanc et al. reported similar occurrences of grade 3/4 toxicities in patients taking either pravastatin, sorafenib, both medications or none of the medications in a study involving mostly Child–Pugh B (96.8%) [43]. Munoz et al. studied simvastatin in 30 patients with decompensated cirrhosis and reported 23% myalgia, 13% myalgia plus creatine kinase increase, 3% new onset diabetes, 63% digestive symptoms, and 13% headache [44] (after the search date of this review, the full article has now been published without changes in these figures [45]). In a retrospective case-controlled study, Singh et al. evaluated the safety of statins in users compared to non-users with decompensated cirrhosis and reported one case of muscle injury in statin user group (*n* = 195) [46]. The rate of non-liver transplant hospitalization was similar between the two groups (62.1% in statin group vs. 62.2% in non-statin group) [46].

### Dose adjustment methods of statins in cirrhosis

After systematically searching all available evidence associated with cirrhosis and statin medication in the prespecified databases, we could not identify any study addressing if any dose adjustment method is superior for dosing statins in cirrhosis.

### Quality Assessment

Summary of quality assessment of the included studies can be found in Additional file 1: Table S3 and Additional file 1: Table S4. RCTs were assessed with Cochrane Risk of Bias tool [24] and observational studies were assessed with Newcastle–Ottawa Quality Assessment Scale [25]. Note that pharmacokinetic studies, review article, drug product monographs and abstracts were not assessed as the tools used did not apply to them.

## Discussion

### Pharmacokinetics of statins in cirrhosis

An understanding of pharmacokinetic changes of statins in the setting of cirrhosis can be useful in predicting the likelihood of potential adverse effects, and could inform statin dose adjustments. Unfortunately, the available evidence to answer this question is limited. Many of the resources identified in this review were secondary and tertiary literature such as review articles and drug product monographs.

Since statins undergo extensive first-pass effect (with the exception of pitavastatin), CYP450 metabolism, and bile excretion [17, 20], the presence of cirrhosis with liver dysfunction and portal systemic shunting are expected to interfere with the pharmacokinetics of statins. There was a general trend of increase in both AUC and Cmax of different statins in patients with Child–Pugh classes A and B compared to non-cirrhotic patients. In addition, the degree of increase of the two parameters appeared to be dependent upon the severity of liver impairment, with higher AUC and *C*_max_ in Child Pugh B compared to Child Pugh A. The two exceptions, where the results of AUC and *C*_max_ were not statistically significant, were pitavastatin [36] in Child–Pugh A and rosuvastatin [38] in both Child–Pugh A and B. The lower rate of first-pass metabolism [36] and minimal CYP450 metabolism [17, 20, 35, 36] of pitavastatin could potentially explain the only minor, non-significant increase of AUC and *C*_max_ in Child–Pugh A. However, as pitavastatin undergoes biliary excretion [35, 36], this may potentially contribute to larger increases at later stages of hepatic impairment where cholestasis tends to be more severe. [47] Simonson et al. showed that AUC and Cmax of rosuvastatin did not differ between Child–Pugh A and B patients and healthy individuals, although higher values of both parameters were observed in two individuals with the highest Child–Pugh scores [38]. The authors suggested that the changes in the hepatic OATP expression associated with hepatic impairment that altered the liver uptake of rosuvastatin which resulted in lower first pass effect and higher systemic bioavailability [38]. Indeed, the expression of multiple SLC and ABC transporters are altered by the presence of cirrhosis [21, 22]. Interestingly, the etiology of cirrhosis may influence the levels of transporters [22]. For example, Wang et al. reported hepatic OATP 1B1 is decreased in alcoholic cirrhosis but not in hepatitis C induced cirrhosis. [22] Furthermore, genetic polymorphism also influences the expression of the membrane transporters [17, 19]. and therefore adds to the complexity when interpreting pharmacokinetic changes of statins in the setting of cirrhosis. Similar to pitavastatin, rosuvastatin also undergoes limited liver metabolism and is largely excreted in bile [20, 38]. Again, these could potentially explain the mild effect on AUC and *C*_max_ in patients with cirrhosis. One other difference between rosuvastatin and pitavastatin is the lipophilicity. Rosuvastatin is hydrophilic and therefore, more hepatoselective and relies on active carrier-mediated process for liver uptake, rather than passive diffusion in lipophilic statins such as pitavastatin [20]. Overall, the degree of increase in AUC and *C*_max_ associated with pitavastatin and rosuvastatin is relatively mild compared to fluvastatin and atorvastatin. Atorvastatin showed the largest increase in both AUC and *C*_max_ in patients with Child–Pugh A and B classes [34]. We could not find pharmacokinetic information on simvastatin and lovastatin in cirrhosis.

Overall these studies had significant limitations. Sample sizes were small, as reflected by the wide confidence intervals [36, 38]. In addition, single-dose pharmacokinetic studies do not provide useful information on efficacy and safety of statins, so it is unknown if changes in AUC and *C*_max_ have clinical relevance. Indeed, none of the studies reporting clinical outcomes or safety of statins in cirrhosis provided pharmacokinetic information. These RCTs focused on liver-specific efficacy outcomes including hepatic venous pressure gradient (HVPG] [42, 48–50], mortality and survival [33, 42, 51], survival in hepatocellular carcinoma [31, 52], variceal bleeding [33, 42, 51], and liver stiffness [53].

### Cardiovascular efficacy of statins in cirrhosis

Several systematic reviews previously reported on the relationship between statin use and liver-specific endpoints and therefore this was not further addressed here [9–11]. Systematic reviews have clearly shown the efficacy of statins on cardiovascular outcomes in populations without cirrhosis [7, 54]. However, to our knowledge, this is the first systematic review that explores the relationship between statin use and cardiovascular outcomes specifically in patients with cirrhosis. This is an important clinical question since it is predicted that the prevalence of compensated cirrhosis due to nonalcoholic fatty liver disease (NAFLD) will increase by 93% between 2019 and 2030 in Canada [16]. Patients with NAFLD have multiple cardiovascular risk factors and are at higher risk of cardiovascular morbidity and mortality [55]. Since statins might be associated with a higher rate of adverse effects in cirrhosis, even if these are infrequent it is crucial to balance them with the degree of statin cardiovascular benefits in this population. Unfortunately, none of the RCTs of statins in cirrhosis reported on cardiovascular outcomes. Only one retrospective cohort study reported MACE [40]. Noticeably, it showed a lower rate of MACE in non-[statin] initiators compared to existing [statin] users, and this was probably due to unbalanced prognostic factors including lower incidence of diabetes mellitus, pre-existing coronary artery disease, obesity, and NAFLD or NASH induced cirrhosis in the non-[statin] initiator group [40].

### Safety of statins in cirrhosis

For safety outcomes in RCTs, there were significant variations in terms of the statins being studied, etiology and severity of cirrhosis, comorbidities, and the specific safety endpoints being measured. Five major safety outcomes were reported in this review (Table 1); these were composite outcomes of rhabdomyolysis or muscle injury, rhabdomyolysis, muscle injury, myalgia, and drug-induced liver injury. Drug-induced liver injury was of difficult interpretation due to the confounding of concomitant muscle injury, which leads to an increase in aminotransferases [56]. The pooled estimate of rhabdomyolysis or muscle injury with simvastatin 40 mg was 4%, while it was 2% for rhabdomyolysis. In comparison, risk of rhabdomyolysis with any statin use in the general population was estimated to be 0.01% [57]. Moreover, in the Heart Protection Study, where patients with cirrhosis were excluded, the incidence of rhabdomyolysis with simvastatin 40 mg daily was 0.05% over an average follow-up of 5 years [13]. Thus, based on the findings of this systematic review, there is approximately a 40-fold increased risk of rhabdomyolysis among cirrhosis patients taking simvastatin 40 mg. Even when comparing to simvastatin 80 mg, in which the SEARCH study showed 0.1% of confirmed rhabdomyolysis over a mean follow-up of 6.7 years [58], the risk of rhabdomyolysis in cirrhosis patients on simvastatin 40 mg is still 20 fold higher. It is important to note that participants who developed rhabdomyolysis had either advanced or decompensated cirrhosis (Child–Pugh B or C) and the study reporting the highest rate of rhabdomyolysis was the one including more advanced patients [59]. Therefore, it would be difficult to conclude if the same risk can be translated into patients with compensated cirrhosis. The pooled point estimate for myalgia was 6%, although there was significant heterogeneity among the studies likely due to its subjectivity and differences on the methodology of the trials to assess this specific symptom.

Rhabdomyolysis was not reported in simvastatin 20 mg or other statins (Table 1) but this observation is based in a very low number of patients. For simvastatin 20 mg, there were only 2 studies with a total of 52 participants. Assuming the true underlying risk of rhabdomyolysis of simvastatin 20 mg is the same as simvastatin 40 mg (2%), the probability of not finding any case after 52 participants is 35% (according to a binomial distribution). With an even lower true underlying risk, such as 1.5%, this probability would be 46%. Therefore, there is still substantial uncertainty about the muscle safety of simvastatin 20 mg in decompensated cirrhosis, that may be clarified by a long-term randomized trial in patients with decompensated cirrhosis (NCT03780673 [60]). As mentioned previously, atorvastatin was noted to have the highest increase of AUC and Cmax, implying higher exposure. Logically one would expect patients with cirrhosis to develop more adverse effects. However, this was not seen in the two RCTs included. [42, 53] It is important to note that both studies were assessed to be at high risk of bias and low quality (Additional file 1: Table S3). In conclusion, with the reported risk of rhabdomyolysis and muscle injury, alternatives to simvastatin 40 mg should be considered for studies in decompensated cirrhosis. At present, the scarcity of safety data in other statins limits the selection of a safer statin. The lack of both safety information and efficacy data in terms of prevention of cardiovascular risk (a significant cause of morbidity in NAFLD cirrhosis), calls for specific studies addressing these issues to guide clinical decisions. Despite the encouraging data suggesting a potential benefit for statins in cirrhosis [8–11, 61, 62], until new safety data is available, statins should not be used routinely in decompensated cirrhosis, and if used, it should be under close monitoring of muscle toxicity.

### Guiding dose adjustment of statins in cirrhosis

We did not identify any studies comparing different methods for adjusting statin dose in cirrhosis. There are few possible reasons for this. First, there are no definitive global methods of medication dose adjustment used in cirrhosis. Unlike in renal impairment, where creatinine clearance or serum creatinine is used for dose adjustment, there is not a single reliable marker that allows the same in patients with liver function impairment [1]. While FDA recommends manufacturers to study drugs in different Child–Pugh classes to enable dosing recommendations in hepatic impairment [3]. The product monographs of some statins do not contain hepatic dose adjustment information and list active liver disease as a contraindication [34, 35]. Clearly, there is a need for evidence-based hepatic dose adjustment methods for all medications including statins. Such method(s), when available, should not only reveal equivalent pharmacokinetic parameters in a short term but also demonstrate comparable efficacy and safety endpoints in the long run.

## Strengths and limitations

There are several strengths in this systematic review. First, we conducted a comprehensive search in multiple databases in an attempt to capture all relevant studies related to cirrhosis and statins. Second, this single systematic review aimed to address three important clinical questions. Even though we were unable to identify any information to answer the last question, we show here the paucity of evidence in this area, which in turn may be helpful to guide future research.

There are also limitations in this systematic review. Many of the studies included were at high risk of bias and low overall quality. This could affect the validity of the results and therefore lead to incorrect conclusions. In addition, the pharmacokinetic data identified were, in part, from product monographs and one review article. There is a clear scarcity of primary literature in this field. As a result, we were unable to perform a quantitative meta-analysis on this question.

## Summary and conclusions

In summary, cirrhosis has a minor impact on the pharmacokinetics of pitavastatin and rosuvastatin, whereas the impact on atorvastatin pharmacokinetics is profound. There is a lack of RCTs that investigate statin’s efficacy on cardiovascular outcomes in patients with cirrhosis. The proportion of simvastatin 40 mg users experiencing at least some degree of muscle injury is 4% and rhabdomyolysis is 2%. Rhabdomyolysis was not reported with simvastatin 20 mg or other statins although there were only a few studies available with small sample sizes. Lastly, there is a paucity of data in hepatic dose adjustment methods for statins.

In conclusion, based on the available data, the use of simvastatin 40 mg in the setting of advanced or decompensated cirrhosis should be avoided. Overall, the paucity of evidence presents a major impediment to evidence-based use of statins for CV indications in patients with cirrhosis, and to the need of further studies in this area.

## Supplementary information

**Additional file 1**. Supplementary Data.

**Additional file 2**. Research Protocol.

## Data Availability

All data generated or analysed during this study are included in this published article [and its supplementary information files].

## Abbreviations

ABC transporters: ATP-binding cassette transporters
ALT: alanine aminotransferase
ASA: aspirin
AST: aspartate aminotransferase
AUC: area under the curve
AUC_0-24_: area under the curve from time 0 to 24 h
AUC_inf_: area under the curve extrapolated from time 0 to infinity
bpm: beat per minute
CK: creatine kinase
CL_R_: renal clearance
*C*_max_: maximum plasma concentration
*C*_min_: minimum observed plasma drug concentration
CPA: child–pugh A
CPB: child–pugh B
CPK: creatine phosphokinase
CTP: child turcotte pugh
CV: cardiovascular
CVD: cardiovascular disease
CYP450: cytochrome P450
DM: diabetes mellitus
EMA: European Medicines Agency
EtOH: ethanol
FDA: Food and Drug Administration
F_e_: fraction of (rosuvastatin) excreted in the urine as unchanged drug
GI: gastrointestinal
HBV: hepatitis B virus
HCV: hepatitis C virus
HMG-CoA: hydroxymethylglutaryl-CoA
HR: hazard ratio
HTN: hypertension
HVPG: hepatic venous pressure gradient
INR: international normalized ratio
LDL: low-density lipoproteins
MACE: major adverse cardiovascular events
MDR1: multidrug resistance protein 1
MELD/Na: model for end-stage liver disease sodium
MELD: model for end-stage liver disease
MI: myocardial infarction
N/A: not applicable
NASH: nonalcoholic steatohepatitis
NS: non-significant
NSBBs: non-selective beta-blockers
NYHA: New York Heart Association
OATP: organic anion transporting polypeptide
PK: pharmacokinetics
PK: pharmacokinetics
PSC: primary sclerosing cholangitis
PT: prothrombin time
RCT: randomized controlled trial
SBP: systolic blood pressure
SGPT: serum glutamic-pyruvic transaminase
SLC membrane transporters: solute carrier membrane transporters
*t*_1/2_: half-life
TIPS: transjugular intrahepatic portosystemic shunt
*T*_max_: time of *C*_max_ (maximum plasma concentration)
ULN: upper limit of normal
Vd: volume of distribution
