# Supplementary material for: A systematic review on pharmacokinetics, cardiovascular outcomes and safety profiles of statins in cirrhosis
Source: BMC Gastroenterol. 2021 Mar 16;21:120. doi: 10.1186/s12876-021-01704-w (PMC7967963; doi:10.1186/s12876-021-01704-w)
Supplement: Supplementary file 1 — Additional file 1. Supplementary Data. [file 12876_2021_1704_MOESM1_ESM.docx]

**A Systematic Review on Pharmacokinetics, Cardiovascular Outcomes and Safety Profiles of Statins in Cirrhosis**

Shuen Sung^a^, Mustafa Al-Karaghouli^a^, Sylvia Kalainy^b^, Lourdes Cabrera Garcia^c^, Juan G Abraldes^a^

^a^Faculty of Medicine & Dentistry, University of Alberta, Edmonton, Alberta, Canada;

^b^Alberta Health Services, Edmonton, Alberta, Canada;

^c^Faculty of Medicine, Complutense University of Madrid, Madrid, Spain

**Supplementary Data**

Supplementary Table 1: Data extraction for pharmacokinetics outcomes

| **Authors/ Publication year** | **Study type** | **Number of participants/**  **duration of study** | **Participant characteristics** | **Inclusion/ exclusion criteria** | **Intervention** | **PK outcomes** | **Safety outcomes** |
| --- | --- | --- | --- | --- | --- | --- | --- |
| Hui 2005 (36) | Pharmacokinetics | n=18  Single-dose study  Duration: 72 hours | All male; 6 with Child-Pugh A, median age 42.5; 6 with Child-Pugh B median age 46.5; 6 control median age 37.5 | Exclusion: esophageal variceal bleeding in the past 6 months, portosystemic shunt, INR>2.0, bilirubin ≥190µmol/l; transaminases >10x ULN; use of medications that can affect hepatic metabolism in the past month; use of herbal medications in the past week | Pitavastatin single dose of 2mg given after overnight fasting.  Blood samples are taken at various interval to measure plasma concentration of pitavastatin (P) and pitavastatin lactone (PL) | **Geomean ratio of P:**  Cmax: 1.19 (0.74-1.90) in CPA; 2.47 (1.39-4.39) in CPB (p=0.010)  AUC_inf_: 1.28 (0.66-2.49) in CPA; 3.54 (1.82-6.88) in CPB (p=0.002)  t_1/2_: 1.18 in CPA; 2.06 in CPB (p=0.06)  **Geomean ratio of PL:**  Cmax: 0.94 in CPA; 0.49 in CPB (p=0.008)  AUC_inf_: 0.85 in CPA (*NS*); 0.76 in CPB (*NS*)  t_1/2_: 0.85 in CPA (*NS*); 1.06 in CPB (*NS*) | N/A |
| Smith 1993 (37) | Pharmacokinetics | n=22  Single-dose study | Not reported (n=11 in hepatic insufficiency group, n=11 in healthy volunteers, age and gender-matched) | Inclusion: age 21 to 65; Child-Pugh<10 (no individual item >2); serum transaminase and total bilirubin <3x ULN | Fluvastatin 40mg as a single dose in the morning | Hepatic insufficiency vs. healthy volunteers:  AUC: 795ng h/mL vs. 304ng h/mL  C_max_: 683ng/mL vs. 269ng/mL  No difference in mean t_max_ parameters and terminal half-lives.  Plasma clearance is ~ 28% less and Vd is ~31% smaller in hepatic insufficiency | N/A |
| Simonson 2003 (38) | Pharmacokinetics  non-randomized, open-labelled, parallel group | n=18  Duration: 18 days | **n=18:** mean age: 51; male: 67%;  **normal hepatic function (n=6)**: AST: 21.0U/L; ALT: 23.7U/L; total bilirubin: 9.2µmol/L; albumin: 42.0g/L;  **mild hepatic impairment CPA (n=6)**: AST: 29.7U/L; ALT: 25.5U/L; total bilirubin: 14.5µmol/L; albumin: 42.5g/L;  **moderate hepatic impairment CPB (n=6)**: AST: 40.5U/L; ALT: 34.8U/L; total bilirubin: 24.5µmol/L; albumin: 39.7g/L;  Etiology of cirrhosis in hepatic impairment: alcohol | Subjects of normal hepatic function and hepatic impairment were included. | Rosuvastatin 10mg daily at 0700 for 14 days; | **normal vs. CPA vs. CPB:**  AUC_0-24_(ng h/ml): 60.7 vs 63.7 vs. 73.3  -ratio of geomeans of CPA to normal: 1.05 (0.58 to 1.91);  -ratio of geomeans of CPB to normal: 1.21 (0.51 to 2.84)  C_max_(ng/ml): 6.02 vs. 9.29 vs. 12.8  -ratio of geomeans of CPA to normal: 1.54 (0.94 to 2.52);  -ratio of geomeans of CPB to normal: 2.13 (0.65 to 6.95)  C_min_(ng/ml): 0.7 vs. 0.7 vs. 0.6  T_max_(h): 3.50 vs. 1.50 vs. 2.50  CL_R_ (ml/min): 153 vs. 123 vs. 106  F_e_ (%): 5.81% vs. 4.91% vs. 4.85% | Well tolerated by all participants, no serious adverse effects, no withdrawals. |
| Lipitor® monograph (34) | Product monograph | N/A | N/A | N/A | Atorvastatin | Child-Pugh A:  C_max_: 4 fold increase  AUC: 4 fold increase  Child-Pugh B:  C_max_: 16 fold increase  AUC: 11 fold increase | N/A |
| Livalo® monograph (35) | Product monograph | N/A | N/A | N/A | Pitavastatin | Child-Pugh A (mild hepatic impairment):  C_max_: 30% higher (1.3 fold)  AUC_inf_: 60% higher (1.6 fold)  T_1/2_: 10 hours (healthy volunteers: 8 hours)  Child-Pugh B (moderate hepatic impairment):  C_max_: 2.7 fold increase  AUC_inf_: 3.8 fold increase  T_1/2_: 15 hours (healthy volunteers: 8 hours) | N/A |
| Wright 2015 (39) | Review | N/A | N/A | N/A | Pravastatin | Cirrhosis (CTP class not specified):  Cmax: 1.34 fold  AUC: 1.52 fold | N/A |

Note: only relevant inclusion and exclusion criteria were extracted.

N/A: not applicable; CPA: Child-Pugh A; CPB: Child-Pugh B; PK: pharmacokinetics; AUC: area under the curve; AUC_inf_: area under the curve extrapolated from time 0 to infinity; Cmax: maximum plasma concentration; t_1/2_: half-life; NS: non-significant; INR: international normalized ratio; ULN: upper limit of normal; ASA: aspirin; ALT: Alanine aminotransferase; AST: Aspartate aminotransferase; AUC_0-24_: area under the curve from time 0 to 24 hours; C_min_: minimum observed plasma drug concentration; Vd: volume of distribution; CL_R_: renal clearance; F_e_: fraction

of (rosuvastatin) excreted in the urine as unchanged drug; T_max_: time of Cmax (maximum plasma concentration)

Supplementary Table 2: Data extraction for Cardiovascular efficacy and safety outcomes

| **Authors/ Publication year** | **Study type** | **Number of participants/**  **duration of study** | **Participant characteristics** | **Inclusion/exclusion criteria** | **Intervention** | **Efficacy outcomes** | **Safety outcomes** |
| --- | --- | --- | --- | --- | --- | --- | --- |
| Abraldes 2009 (48) | RCT | n=59 for safety analysis  n=55 for efficacy analysis  Duration: 30 days | Simvastatin (n=28): mean age 58; 39% female; 64% Child-Pugh A, 36% Child-Pugh B  Placebo (n=27): mean age 56; 22% female; 59% Child-Pugh A, 30% Child-Pugh B; 11% Child-Pugh C | Inclusion:  Exclusion: cholestatic liver disease; severe liver failure: presence of bilirubin level >5 mg/dL, prothrombin rate <40%; Child–Pugh score ≥12; previous surgical shunt or TIPS | Simvastatin 20mg PO daily x 15 days then ↑ 40mg daily x 15 days if no adverse effects vs. Placebo | N/A | **Simvastatin (n=30) vs. placebo (n=29):** participants with adverse events: 3 vs. 7; ↑AST >2x: 1 vs. 1; ↑CK >2x: 2 vs. 1; muscle weakness or myalgias: 1 vs. 1 |
| Abraldes 2016 (51) | RCT | n=149 for safety analysis  n=147 for efficacy analysis  Duration: median follow-up 382 days in plaebo; 371 days in simvastatin | Simvastatin (n=69): mean age 57.42; male: 65.2%; alcoholic cirrhosis: 71%; Hepatitis C cirrhosis: 27.5%; Child Pugh A/B/C %: 15/68/17; MELD mean score: 10.15  Placebo (n=78): mean age 57.62; male: 67.9%; alcoholic cirrhosis: 71.4%; Hepatitis C cirrhosis: 22.1%; Child Pugh A/B/C %: 24/62/14; MELD mean score: 10.03 | Inclusion: index variceal bleeding within the previous 5–10 days  Exclusion: Child-Pugh score >13; pretreatment with portosystemic shunt (surgical or percutaneous); previous treatment with statins within 1 month of randomization. | Simvastatin 20mg PO daily x 15 days then ↑ 40mg daily x 15 days if no adverse effects **vs.** Placebo  All participants were treated with endoscopic variceal ligation and NSBBs for secondary prophylaxis of variceal bleeding. | N/A | **Simvastatin (n=70) vs. placebo (n=79):** any adverse events: 81.4% vs. 75.9%; serious adverse events: 52.8% vs. 55.6%; abdominal pain: 0% vs. 3.8%; rhabdomyolysis: 2.8% vs. 0% |
| Bishnu 2018 (42) | RCT | n=33 recruited  n=23 for final analysis  Duration 1 year | Propranolol (n=12): mean age: 46.67, male: 100%; alcohol-related cirrhosis: 50%; Child-Pugh median score: 6.5; MELD median score: 11  Propranolol + atorvastatin (n=11): mean age: 44, male: 81.82%; alcohol-related cirrhosis: 36.36%; Child-Pugh median score: 6; MELD median score: 11 | Inclusion: Portal hypertension  Exclusion: Child-Pugh C; hepatocellular carcinoma; previous portosystemic shunt surgery; previous episodes of rhabdomyolysis | Propranolol 40mg PO daily **vs.** Propranolol 40mg PO daily + Atorvastatin 20mg PO daily | N/A | No safety data reported |
| Elwan 2018 (63) | RCT | n=40  Duration: 30 days | Simvastatin (n=20): age: 51.5; female: 50%; ascites: 75%; History of upper endoscopy with varices: 80%; % of Child-Pugh A/B/C: 15/60/25; AST 64.9; ALT: 40.3; serum albumin 2.780g/dL; total serum bilirubin: 4.785g/dL  Controlled (n=20): age: 50.8; female: 20%; ascites: 80%; History of upper endoscopy with varices: 70%; % of Child-Pugh A/B/C: 5/45/50; AST 51.75; ALT: 39.4; serum albumin 2.710g/dL; total serum bilirubin: 2.660g/dL | Inclusion:  Exclusion: hepatocellular carcinoma | Simvastatin 20mg PO x 2 weeks then ↑ 40mg PO x 2 weeks **vs.** controlled (did not receive simvastatin) | N/A | **Simvastatin vs. Controlled:**  Myalgia or muscle pain: n=2 (10%) vs. n=4 (20%) (p=0.376)  Diarrhea: n=2 (10%) vs. n=1 (5%) (p=0.548)  Worsening of ascites: n=3 (15%) vs. n=5 (25%) (p=0.429) |
| Ghadir 2019 (53) | RCT | n=40  Duration: 6 months | Atorvastatin + ASA (n=16): mean age: 50.3; male: 75%; SGOT (AST): 44.87; SGPT (ALT): 44.12; Child-Pugh score: 7.50  Atorvastatin + Placebo (n=17): mean age: 47.9; male: 82%; SGOT (AST): 39.05; SGPT (ALT): 41.76; Child-Pugh score: 6.59 | Inclusion: LDL >70mg/dL;  Exclusion: 3x ULN of liver transaminases 3 months after start of trial; noncompliance during follow-up; statin induced myopathy, current use of fibrates, history of GI bleeding or esophageal  varices, liver stiffness≥ F3 in Fibroscan or stigmata of recent gastroesophageal variceal bleeding. | Atoravastatin 20mg daily + ASA 80mg daily **vs.** Atorvastatin 20mg daily + Placebo daily | N/A | Atoravastatin + ASA **vs.** Atorvastatin + Placebo:  3x ULN of liver transaminases 3 months or noncompliance: 4 vs. 3 (these participants were excluded from final analysis); SGOT (AST): 43.75 vs. 40.24; SGPT (ALT): 44.94 vs. 47.35; |
| Jha 2019 (33) | RCT | n= 164; n=134 (30 participants excluded after randomization  Duration: mean follow up: 49.05 weeks | Carvedilol (n=69): mean age: 46.02; male: 73.9%; Etiology of cirrhosis: 36% alcohol; 33% hepatitis B; 20% cryptogenic; MELD/Na mean score: 16.49; CTP mean score: 8.52; CTP (A/B/C)%: 13/62.4/24.6; ascites: 81.2%; mean albumin 2.66g/dL; mean ALT 54IU/L;  Carvedilol + simvastatin (n=65): mean age: 44.86; male: 69.2%; Etiology of cirrhosis: 26% alcohol; 29% hepatitis B; 28% cryptogenic; MELD/Na mean score: 15.03; CTP mean score: 8.29; CTP (A/B/C)%: 9.2/70.8/20; ascites: 81.5%; mean albumin 2.78g/dL; mean ALT 55IU/L; | Inclusion: history of hematemesis and/or melena due to esophageal varices 5 to 10 days prior to randomization;  Exclusion: non-cirrhotic cause of variceal bleeding; history of previous shunt surgery or TIPS procedure; hepatocellular carcinoma; bleeding due to gastric varices; history of myopathy | Carvedilol 6.25mg daily and ↑ 12.5mg daily over 7 days if tolerated. Dose reduced if SBP <90mmHg, HR <55bpm or other adverse effects **vs.** Simvastatin 20mg daily and ↑ 40mg daily after 7 days if tolerated + Carvedilol 6.25mg daily and ↑ 12.5mg daily over 7 days if tolerated. Dose reduced if SBP <90mmHg, HR <55bpm or other adverse effects | N/A | Authors did not find simvastatin induced significant adverse effects that required drug discontinuation; no significant elevation of CPK level in simvastatin group. |
| Jouve 2019 (52) | RCT | n=323  Median duration: 4.1 months in sorafenib-pravastatin group; 3.6 months in sorafenib group. | Median age 68; male: 92.3%; **associated cirrhosis: 84.8%**; esophagel varices: 34.4%; splenomegaly: 23.8%  mean age: 65 (estimated using formula) | Inclusion: HCC, Child-Pugh score A, liver aminotransferases ≤5x ULN;  Exclusion: Recent or present life-threatening extrahepatic disease; cardiac disease (Heart Failure NYHA class≥2, uncontrolled HTN or arrhythmia, MI <6 months); | Sorafenib 400mg BID + pravastatin 40mg daily **vs.** sorafenib 400mg BID | N/A | Sorafenib + pravastatin vs. sorafenib:  Digestive toxicity: Grade 1-2: 77.4% vs. 74.5%, Grade 3-4: 16.1% vs. 15.9%;  Abdominal pain: Grade 1-2: 34.8% vs. 38.9%, Grade 3-4: 7.1% vs. 6.4%;  Muscular pain: Grade 1-2: 15.5% vs. 12.1%, Grade 3-4: 1.9% vs. -;  Hepatobiliary disorders: Grade 1-2: 94.2% vs. 97.5%, Grade 3-4: 67.1% vs. 52.9% |
| Pollo-Flores 2015 (49) | RCT | n=34  Duration: 3 months | Simvastatin (n=14): median age: 56.5; male: 57%; Child-Pugh median score: 6; Child-Pugh A/B/C: 8/5/1; median MELD score: 10; median albumin: 3.4g/L; median total bilirubin: 1.1mg/dL; median INR: 1.3; median ALT: 72.5U/L; ascites: 21%;  Placebo (n=20): median age: 58.5; male: 50%; Child-Pugh median score: 6; Child-Pugh A/B/C: 14/5/1; median MELD score: 10.5; median albumin: 3.5g/L; median total bilirubin: 1.1mg/dL; median INR: 1.3; median ALT: 64.5U/L; ascites: 25%; | Inclusion: portal hypertension  Exclusion: aminotransferases levels >3x above ULN; within the last 6 months or current use of simvastatin; hepatocellular carcinoma; decompensated cirrhosis; | Simvastatin 20mg daily x 2 weeks, then ↑ 40mg daily **vs.** Placebo | N/A | Simvastatin vs. Placebo:  myalgia: 7% vs. 10% p=0.90; diarrhea: 7% vs. 5% p=0.93; chest pain: 7% vs. 0 p=0.74; epistaxis: 7% vs. 0 p=0.74;  ALT decreased in simvastatin group but did not reach significance. |
| Pose 2020 (59) | RCT | n= 50 (n=44 for full analysis)  Duration: median follow-up: 84 days | Simvastatin 40mg (n=16): mean age: 60; female: 25%; alcohol induced cirrhosis: 56%; bilirubin: 2.5mg/dL; albumin 32g/L; Child Pugh B: 75%; Child Pugh C: 25%; mean MELD score: 14  Simvastatin 20mg (n=14): mean age: 49; female: 21%; alcohol induced cirrhosis: 64%; bilirubin: 2.7mg/dL; albumin 33g/L; Child Pugh B: 71%; Child Pugh C: 29%; mean MELD score: 14  Placebo (n=14): mean age: 59; female: 36%; alcohol induced cirrhosis: 64%; bilirubin: 2.1mg/dL; albumin 34g/L; Child Pugh B: 71%; Child Pugh C: 29%; mean MELD score: 13 | Inclusion:  Exclusion: patients with  acute-on-chronic liver failure; INR>2.5; creatine kinase ≥ 50% above ULN at inclusion; hepatocellular  carcinoma outside Milan criteria; | Simvastatin 40mg daily + Rifaximin 1200mg daily **vs.** Simvastatin 20mg daily + Rifaximin 1200mg daily **vs.** placebo of both medications | N/A | Simvastatin 40mg + Rifaximin group:  AST - mean difference vs. placebo: 130IU/L (54 to 205) p=0.0009;  ALT -mean difference vs. placebo: 61 IU/L (22 to 100) p=0·0025;  AST - mean difference vs. simvastatin 20mg: 143IU/L (66 to 220) p=0.0003;  ALT -mean difference vs. simvastatin 20mg: 69 IU/L (29 to 109) p=0.0009;  CK - mean difference vs. placebo: 1009 IU/L (208 to 1809) p=0·014.  CK - mean difference vs. simvastatin 20mg: 1004 IU/L (192 to 1817) p=0·016;  Rhabdomyolysis n=3  Simvastatin 20mg + Rifaximin group:  AST - mean difference vs. placebo: –14 IU/L (–91 to 64) p=0·728; ALT - mean difference vs. placebo: –8 IU/L  (–49 to 33); p=0·698;  CK - mean difference vs. placebo: (4·2 IU/L (–804 to 813) p=0·992; |
| Riaño 2020 (31) | RCT | n=32 (n=31 for analysis)  Duration: 177.6 days in controlled group; 251.2 days in treatment group | Mean age: 61.4; cirrhosis: 90.3%; male: 93.5%; Child-Pugh A/B: 90.3%/9.7%; mean albumin 38.83g/L; INR 1.16; total bilirubin ≤1.2mg/dL: 61.3%, >1.2mg/dL: 35.5%; | Inclusion: HCC; Child-Pugh class A or B7;  Exclusion: liver transplantation; heart failure NYHA >class II; uncontrolled arterial hypertension; uncontrolled arrhythmias; acute MI in previous 6 months; major hemorrhagic diseases; | Sorafenib 400mg BID + pravastatin 40mg daily **vs.** sorafenib 400mg BID + placebo | N/A | Sorafenib + pravastatin vs. sorafenib + placebo (number of adverse events): GI disorders: 27 vs. 39; diarrhea: 9 vs. 11; abdominal pain: 8 vs. 6; |
| Vijayaraghavan 2020 (50) | RCT | n=220 (n=163  Duration: 3 months | Carvedilol (n=110): mean age: 52.5; etiology of cirrhosis: alcohol 35.5%, NASH 38.2%, HBV 9.1%, HCV 11.8%; Child Pugh A/B/C: 43.6%/38.2%/18.2%; mean CTP score: 7.29; mean MELD score: 13.9; ascites: 21.8%; mean serum bilirubin: 2.36mg/dL; mean AST: 68.85IU/L; mean ALT: 44.46IU/L; mean albumin: 3.27g/dL; INR: 1.56;  Carvedilol + Simvastatin (n=110): mean age: 51.1; etiology of cirrhosis: alcohol 40%, NASH 43.6%, HBV 6.4%, HCV 5.5%; Child Pugh A/B/C: 33.6%/50.9%/15.5%; mean CTP score: 7.29; mean MELD score: 13.9; ascites: 21.8%; mean serum bilirubin: 2.36mg/dL; mean AST: 68.85IU/L; mean ALT: 44.46IU/L; mean albumin: 3.27g/dL; INR: 1.56; |  | Carvedilol 3.125mg BID ↑ 12.5mg BID in 3 weeks if tolerated **vs.** Carvedilol (same dosing increasing) + Simvastatin 20mg QHS ↑ 40mg QHS if tolerated at day 15 | N/A | Carvedilol vs. Carvedilol + Simvastatin:  lethargy/weakness: 3.7% vs. 17.3% p=0.03; muscle pain: 0% vs. 3.7%; AST/ALT >20x ULN: 0% vs. 3.7%; worsening ascites: 18.3% vs. 14.8% p=0.55;  Rhabdomyolysis: 3 participants suspected to experience rhabdoymolysis in simvastatin group. (same participants that experienced AST/ALT >20X and muscle pain as listed above) |
| Kaplan 2019 (40) | Observational | n=74,984 (n=21,921 existing statin user; n=44,269 statin naïve and non-initiator; n=8794 statin naïve and new initiator) | Existing statin user: median age 64, male: 97.5%, follow-up days 900; etiology of cirrhosis: NAFLD/NASH 23.5%, EtOH 38.6%, HCV 11.2%; DM 70.8%, pre-existing CAD 42.2%; median MELD/Na: 9; CTP A/B/C%: 70.7/26.7/2.6;  Non-initiator: median age 60, male: 97.1%, follow-up days 1068; etiology of cirrhosis: NAFLD/NASH 10.8%, EtOH 28.3%, HCV 19.7%; DM 37.3%, pre-existing CAD 6.4%; median MELD/Na: 9; CTP A/B/C%: 64.2/30.5/5.3;  New initiator: median age 61, male: 97.6%, follow-up days 1905; etiology of cirrhosis: NAFLD/NASH 15.5%, EtOH 33.9%, HCV 15.8%; DM 56.9%, pre-existing CAD 16.9%; median MELD/Na: 9; CTP A/B/C%: 78.5/19.5/2.0; | Inclusion: Newly diagnosed cirrhosis  Exclusion: <180 days of clinical follow-up; development of HCC in <180 days from diagnosis of cirrhosis; Fibrosis-4 score <1.45 | Statin exposure normalized to simvastatin-equivalent doses. Statin dose quantified as ≤20mg/day or > 20mg/day simvastatin-equivalent dose. | Non-initiator vs. existing user:  MACE: unadjusted HR 0.58 (95% CI 0.54-0.62) P<0.0001;  New-initiator vs. existing user:  MACE: unadjusted HR 1.49 (95% CI 1.38-1.61) P<0.0001;  Exisiting user statin-exposure per year:  MACE: HR 0.976 (0.915-1.041) p=0.46  Statin naive group - propensity matched, statin-exposure per year:  MACE: HR 1.039 (1.010-1.069) p=0.008  Impact of 90-day statin exposure on MACE:  Existing users: HR 1.487 (1.218-1.815) P<0.0001;  Statin naive group: HR 1.513 (1.290-1.774) P<0.0001; | N/A |
| Munoz 2019 (32) | Non-randomized | n=18  Duration: simvastatin group: 87.0 months  standard-therapy group: 24.2 months | Simvastatin group: mean age: 63; 66% female; etiology of cirrhosis (n): HCV 4, alcohol 1, NASH 1; Child-Pugh A/B/C (n): 4/4/1; mean MELD score: 10.8; DM: n=5, HTN:n=4; current smoking: n=3; metabolic syndrome: n=2;  Standard-therapy group: mean age: 66; 66% female; etiology of cirrhosis (n): HCV 4, alcohol 1, NASH 1; Child-Pugh A/B/C (n): 4/4/1; mean MELD score: 11.8; DM: n=4, HTN: n=2; current smoking: n=3; metabolic syndrome: n=2; | Inclusion:  Exclusion: | Simvastatin 10 to 40mg/day vs. standard therapy | No cardiovascular events in either group. | No adverse effects and/or serious adverse effects in simvastatin group. |
| Patel 2018 (41) | Observational | n=228 (n=84 that was diagnosed with CAD and included in safety analysis.)  Follow-up: 6 month | Mean age: 57; male 64.5%; DM: 32%; HTN: 42.5%; obesity: 46.1%; dyslipidemia: 26.3%; etiology of cirrhosis: HCV 47.8%, NASH: 23.2%; EtOH: 19.7%; any CAD: 36.8% | Inclusion: decompensated cirrhosis evaluated for liver transplant  Exclusion: no coronary angiography as part of liver transplant evaluation; taking ASA but not for CVD prophlaxis; | Statin (n=19) vs. No statin (n=65) after diagnosis of CAD by angiography | Risk of hospitalization:  aspirin group (n=30) **vs.** statin group (n=19) **vs.** no aspirin/statin group (n=48): 20% vs. 32% vs. 25%, p=0.26 | No significant increase in serum AST, ALT, bilirubin, or MELD score up to 6 months of statin therapy |
| Wani 2017 (64) | Non-randomized | n=102 (n=38 for simvastatin group)  Duration: 4.5 months (3 months with carvedilol, 1.5 months with simvastatin added) | n=38 for those who started on simvastatin:  Mean age: 58.45; female (n): 17; Child-Pugh A/B/C (n): 14/13/11; etiology of cirrhosis (n): alcohol 12, viral 15, NASH or cryptogenic 11; ascites (n): 17; total bilirubin: 2.042mg/dL; serum albumin 3.203mg/dL; PT: 14.105; INR: 1.318 | Inclusion: evidence of esophageal varices on upper gastrointestinal endoscopy, without previous history of hemorrhage and a baseline HVPG > 12mmHg  Exclusion: INR >2.5 or PT < 40% of control; bilirubin >5mg/dL; Hepatocellular carcinoma; previous  surgical shunt or TIPS; treatment with statin in the past 3 months | Carvedilol for 3 months, if no response (n=38), add simvastatin 20mg daily for 15 days, if tolerated then ↑40mg daily for 1 month; | N/A | After 15 days of simvastatin 20mg: one patient with CPK >5x ULN with normal ALT; one patient developed hepatic encephalopathy; one patient developed severe dizziness. |
| Blanc 2018 (43) | Abstract (randomized, open-label, four-arm parallel) | n=157 | Participants with Child-Pugh B (96.8%) with HCC; | Inclusion:  Exclusion: | Sorafenib 800mg daily **vs**. Pravastatin 40mg daily **vs.** Pravastatin 40mg daily + Sorafenib 800mg daily **vs.** best supportive care | N/A | Safety profile of pravastatin were similar to those observed in Child A patients. |
| Munoz 2018 (44)  (Munoz 2020 (45) full article published after initial search) | Abstract (open, uncontrolled, ambispective) | n=30 | decompensated cirrhosis, age: 57; male 67%; etiology of cirrhosis: alcohol 60%; MELD 12.3; | Inclusion:  Exclusion: | Simvastatin 20mg daily x 2 weeks, then ↑ 40mg daily | N/A | myalgia 23%; myalgia plus creatine kinase increase 13%; new onset diabetes 3%; digestive symptoms 63%; headache 13%; liver injury none.  No serious adverse effect or discontinuation. |
| Singh 2019 (46) | Abstract (Retrospective case-controlled study) | n=1191 | decompensated cirrhosis, age: 54.8, male 66.4%, MELD 20.0, CAD 12.3%, DM 32.8%, HTN 44.2%, dyslipidemia 23.8%, alcoholic liver disease 27.5%, HCV 18.0%, NAFLD/NASH 30.0%, PSC 10.7%,  Non-statin users n=996 (84%);  Statin users n=195 (16%): atorvastatin n=104 (53.3%), simvastatin n=39 (20%), pravastatin n=37 (19.0%), rosuvastatin n=10 (5.1%), lovastatin n=5 (2.6%) | Inclusion:  Exclusion: | Statin vs. non-statin | N/A | Muscle injury adverse events: 1 case in statin group.  **Statin vs. non-statin:**  Non-liver transplant hospitalization: 62.1% (n=121) vs. 62.2% (n=620) |

Note: only relevant inclusion and exclusion criteria were extracted.

N/A: not applicable; RCT: randomized controlled trial; CPA: Child-Pugh A; CPB: Child-Pugh B; PK: pharmacokinetics; AUC: area under the curve; AUC_inf_: area under the curve extrapolated from time 0 to infinity; Cmax: maximum plasma concentration; NS: non-significant; NSBBs: non-selective beta-blockers; INR: international normalized ratio; ULN: upper limit of normal; TIPS: transjugular intrahepatic portosystemic shunt; ASA: aspirin; ALT: Alanine aminotransferase; AST: Aspartate aminotransferase; SGPT: serum glutamic-pyruvic transaminase; LDL: low-density lipoproteins; MELD: model for end-stage liver disease; MELD/Na: model for end-stage liver disease sodium; CTP: Child Turcotte Pugh; SBP: systolic blood pressure; HR: heart rate; bpm: beat per minute; CK: creatine kinase; NASH: Nonalcoholic steatohepatitis; HBV: Hepatitis B virus; HCV: Hepatitis C virus; EtOH: ethanol; MACE: Major adverse cardiovascular events; DM: diabetes mellitus; HTN: hypertension; HVPG: hepatic venous pressure gradient; PT: Prothrombin time; NYHA: New York Heart Association; MI: myocardial infarction; GI: gastrointestinal; AUC_0-24_: area under the curve from time 0 to 24 hours; C_min_: minimum observed plasma drug concentration; t_1/2_: half-life; SGOT: serum glutamic-oxaloacetic transaminase; CPK: creatine phosphokinase; CVD: cardiovascular disease; Vd: volume of distribution; PSC: Primary sclerosing cholangitis

Supplementary Table 3: Quality assessment for RCTs with Cochrane Risk of Bias Tool:

| **Authors/ Publication year** | **Sequence generation (selection bias)** | **Allocation concealment (selection bias)** | **Blinding of participants and personnel (performance bias)** | **Blinding of outcome assessors (detection bias)** | **Incomplete outcome data (attrition bias)** | **Selective outcome reporting (reporting bias)** | **Other sources of bias** |
| --- | --- | --- | --- | --- | --- | --- | --- |
| Abraldes 2009 (48) | Low Risk | Low Risk | Low Risk | Low Risk | Low Risk | Low Risk | N/A |
| Abraldes 2016 (51) | Low Risk | Low Risk | Low Risk | Low Risk | Low Risk | Low Risk | N/A |
| Bishnu 2018 (42) | Low Risk | Low Risk | High Risk | Unclear | High Risk | Low Risk | N/A |
| Elwan 2018 (63) | Unclear | Unclear | High Risk | Unclear | Unclear | High Risk | Yes |
| Ghadir 2019 (53) | Low Risk | Unclear | Low Risk | Unclear | High Risk | High Risk | N/A |
| Jha 2019 (33) | Low Risk | Unclear | High Risk | High Risk | Low Risk | Low Risk | Yes |
| Jouve 2019 (52) | Low Risk | Unclear | High Risk | High Risk | Low Risk | Low Risk | N/A |
| Pollo-Flores 2015 (49) | Low Risk | Low Risk | Low Risk | Low Risk | Low Risk | Low Risk | N/A |
| Pose 2020 (59) | Low Risk | Low Risk | Low Risk | Low Risk | Low Risk | Low Risk | N/A |
| Riaño 2020 (31) | Low Risk | Unclear | Low Risk | Unclear | Low Risk | Low Risk | N/A |
| Vijayaraghavan 2020 (50) | Low Risk | Low Risk | High Risk | High Risk | High Risk | Unclear | N/A |
| Munoz 2019 (32) | High Risk | High Risk | High Risk | High Risk | Low Risk | High Risk | Yes |
| Wani 2017 (64) | High Risk | High Risk | High Risk | High Risk | Low Risk | High Risk | N/A |

Supplementary Table 4: Quality assessment for observational studies with Newcastle-Ottawa Quality Assessment Scale

| **Author/ Publication year** | **Representativeness of the exposed cohort** | **Selection of the non exposed cohort** | **Ascertainment of exposure** | **Demonstration that outcome of interest was not present at start of study** | **Comparability of cohorts on the basis of the design or analysis** | **Assessment of outcome** | **Was follow-up long enough for outcomes to occur** | **Adequacy of follow up of cohorts** |
| --- | --- | --- | --- | --- | --- | --- | --- | --- |
| Kaplan 2019 (40) | High Risk | Low Risk (1 star) | Low Risk (1 star) | Low Risk (1 star) | Low Risk (2 stars) | Low Risk (1 star) | Low Risk (1 star) | Low Risk (1 star) |
| Patel 2018 (41) | Low Risk (1 star) | Low Risk (1 star) | Low Risk (1 star) | Low Risk (1 star) | High Risk | Low Risk (1 star) | Low Risk (1 star) | Low Risk (1 star) |

**References:**

Please refer to references of the main article.

**Search Strategy**

**MEDLINE:**

1. liver cirrhosis/ or liver cirrhosis, alcoholic/ or liver cirrhosis, biliary/

2. ("Liver cirrhosis" or "cirrhosis" or "liver fibrosis" or "liver failure" or "alcohol liver cirrhosis" or "biliary cirrhosis" or "compensated liver cirrhosis" or "decompensated liver cirrhosis" or "primary biliary cirrhosis").tw,kf.

3. 1 or 2

4. hydroxymethylglutaryl-coa reductase inhibitors/ or atorvastatin/ or lovastatin/ or pravastatin/ or rosuvastatin calcium/ or simvastatin/

5. (statin* or atorvastatin* or lovastatin* or pravastatin* or rosuvastatin* or simvastatin* or fluvastatin* or lipitor or crestor or lescol or zocor or pravachol or mevacor or HMG-CoA reductase inhibitor* or pitavastatin* or livalo or hydroxymethylglutaryl-coa reductase inhibitor*).mp.

6. 4 or 5

7. 3 and 6

**EMBASE**

1. liver cirrhosis/ or alcohol liver cirrhosis/ or biliary cirrhosis/ or compensated liver cirrhosis/ or decompensated liver cirrhosis/ or primary biliary cirrhosis/

2. ("Liver cirrhosis" or "cirrhosis" or "alcohol liver cirrhosis" or "biliary cirrhosis" or "compensated liver cirrhosis" or "decompensated liver cirrhosis" or "primary biliary cirrhosis").tw,kw.

3. 1 or 2

4. hydroxymethylglutaryl coenzyme a reductase inhibitor/ or atorvastatin/ or fluindostatin/ or mevinolin/ or pravastatin/ or rosuvastatin/ or simvastatin/

5. (statin* or atorvastatin* or lovastatin* or pravastatin* or rosuvastatin* or simvastatin* or fluvastatin* or lipitor or crestor or lescol or zocor or pravachol or mevacor or HMG-CoA reductase inhibitor* or pitavastatin* or livalo or hydroxymethylglutaryl-coa reductase inhibitor*).mp.

6. 4 or 5

7. 3 and 6

**Cochrane**

Search Name: June 02_2020 search1_Cochrane

Last Saved: 03/06/2020 16:44:46

Comment:

ID Search

#1 ("statin*" or "atorvastatin*" or "lovastatin*" or "pravastatin*" or "rosuvastatin*" or "simvastatin*" or "fluvastatin*" or "lipitor" or "crestor" or "lescol" or "zocor" or "pravachol" or "mevacor" or "HMG-CoA reductase inhibitor*" or "pitavastatin*" or "livalo" or "hydroxymethylglutaryl-coa reductase inhibitor*"):ti,ab,kw (Word variations have been searched)

#2 MeSH descriptor: [Hydroxymethylglutaryl-CoA Reductase Inhibitors] explode all trees

#3 ("Liver cirrhosis" or "cirrhosis" or "alcohol liver cirrhosis" or "biliary cirrhosis" or "compensated liver cirrhosis" or "decompensated liver cirrhosis" or "primary biliary cirrhosis"):ti,ab,kw (Word variations have been searched)

#4 MeSH descriptor: [Liver Cirrhosis] explode all trees

#5 #1 or #2

#6 #3 or #4

#7 #5 and #6

**SCOPUS**

(TITLE-ABS-KEY("Liver cirrhosis" or "cirrhosis" or "alcohol liver cirrhosis" or "biliary cirrhosis" or "compensated liver cirrhosis" or "decompensated liver cirrhosis" or "primary biliary cirrhosis")) AND ((TITLE-ABS-KEY("statin*" or "atorvastatin*" or "lovastatin*" or "pravastatin*" or "rosuvastatin*" or "simvastatin*" or "fluvastatin*" or "Lipitor" or "crestor" or "lescol" or "zocor" or "Pravachol" or "mevacor" or "HMG-CoA reductase inhibitor*")) OR (TITLE-ABS-KEY("hydroxymethylglutaryl coenzyme a reductase inhibitor*" or "fluindostatin" or "mevinolin" or "pitavastatin*" OR "livalo" OR "hydroxymethylglutaryl-coa reductase inhibitor*")))

**CINAHL Plus**

S1 (MH "Statins+") OR (MH "Atorvastatin+") OR (MH "Fluvastatin") OR (MH "Lovastatin") OR (MH "Pravastatin") OR (MH "Rosuvastatin") OR (MH "Simvastatin") OR (MH "Pitavastatin Calcium")

S2 "statin*" or "atorvastatin*" or "lovastatin*" or "pravastatin*" or "rosuvastatin*" or "simvastatin*" or "fluvastatin*" or "pitavastatin*" or "Livalo" or "lipitor" or "crestor" or "lescol" or "zocor" or "pravachol" or "mevacor" or "HMG-CoA reductase inhibitor*" or "Hydroxymethylglutaryl-CoA Reductase Inhibitor*"

S3 (MH "Liver Cirrhosis+") OR (MH "Liver Cirrhosis, Alcoholic")

S4 "Liver cirrhosis" OR "cirrhosis" OR "alcohol liver cirrhosis" OR "biliary cirrhosis" OR "compensated liver cirrhosis" OR "decompensated liver cirrhosis" OR "primary biliary cirrhosis"

S5 S1 OR S2

S6 S3 OR S4

S7 S5 AND S6
