# Supplementary material for: A systematic review on pharmacokinetics, cardiovascular outcomes and safety profiles of statins in cirrhosis
Source: BMC Gastroenterol. 2021 Mar 16;21:120. doi: 10.1186/s12876-021-01704-w (PMC7967963; doi:10.1186/s12876-021-01704-w)
Supplement: Supplementary file 2 — Additional file 2. Research Protocol. [file 12876_2021_1704_MOESM2_ESM.docx]

**A Systematic Review on Pharmacokinetics, Cardiovascular Outcomes and Safety Profiles of Statins in Cirrhosis**

Shuen Sung, Mustafa Al-Karaghouli, Sylvia Kalainy, Lourdes Cabrera Garcia, Juan G Abraldes

**Background:**

Unlike in renal impairment, where calculated creatinine clearance or estimated glomerular filtration rate (eGFR) can be effectively used for medication dose adjustments, the methods for dose adjustment in hepatic impairment are not as well established.(1) Child-Pugh classification is sometimes used in hepatic dose adjustments but may not accurately reflect the liver's ability to metabolize medications.(1) Child-Pugh classification is also recommended by FDA to use in drug development.(2) However, the efficacy and safety outcomes of statin therapy from using this method remains unclear. In addition, concurrent renal impairment and hepatic toxicity of certain medications can further complicate the process of dose adjustments in patients with liver cirrhosis. Recently, more attention has been focused on statin’s potential hepatic benefits and its use in patients with cirrhosis.(3) Several systematic reviews have been completed to evaluate liver-specific efficacy outcomes of statins in patients with cirrhosis.(4,5,6) However, the traditional cardiovascular benefits of statins in patients with liver cirrhosis is not well understood as this population was often excluded from major statin trials.(7,8,9) This has created a knowledge gap that may lead to some clinicians hesitate from using statins for cardiovascular indications in cirrhotic patients. Therefore, this research project has three objectives. First, we attempt to identify any evidence in pharmacokinetic changes of statins in the setting of liver cirrhosis. Second, we attempt to identify the changes in cardiovascular outcomes and safety profiles of statins in patients with liver cirrhosis in comparison to the general population. Lastly, we attempt to identify any evidence available to support the use of existing dose adjustment methods of statins, including Child-Pugh scores, MELD score, or clinical gestalt.

**Research Questions:**

1. In adult patients, how does liver cirrhosis affect the pharmacokinetic parameters (absorption, distribution, metabolism, excretion) of HMG-CoA reductase inhibitors?
2. In adult patients, how does liver cirrhosis change the cardiovascular efficacy and safety outcomes of HMG-CoA reductase inhibitors?
3. In adult patients with liver cirrhosis taking HMG-CoA reductase inhibitors, what is the evidence of dose adjustment methods including Child-Pugh score, clinical gestalt, and MELD score for efficacy and safety outcomes?

**Rationale for Current Research:**

1. As statins are generally metabolized by the liver,(10) pharmacokinetic changes are likely experienced by patients with liver cirrhosis. Data on these changes could be used to predict potential adverse event rate, and may also be used to develop dose-adjustment methods for statins.
2. Statins have been shown to have major cardiovascular benefits in the general population, and in those with pre-existing cardiovascular conditions.(7,8,9) However, the absolute cardiovascular benefits and potential adverse outcomes are largely unknown in patients with liver cirrhosis. Identification of such evidence can be helpful to inform clinicians when prescribing statins for non-liver indications such as primary and secondary prevention of cardiovascular diseases.
3. There are no definitive ways for dose adjustment of statins in patients with liver impairment. Therefore the current research is to identify if there is evidence for any dose-adjustment methods for statins and if so, to determine the best evidenced-based approach for dose adjustment of statins in patients with liver cirrhosis.

**Inclusion/Exclusion Criteria:**

Research Question 1: In adult patients, how does liver cirrhosis change the pharmacokinetic parameters (absorption, distribution, metabolism, excretion) of HMG-CoA reductase inhibitors?

| Inclusion criteria | Exclusion criteria |
| --- | --- |
| Study types: systematic reviews (only to review and extract relevant references), randomized controlled trials, cohort studies, case-controlled studies, pharmacokinetic studies  Patient demographics: adult patients (>age 18), history of cirrhosis^+^ from any cause, any indications for statins, taking any statins  Outcome of interest:   - Pharmacokinetic outcomes: Changes in absorption, distribution, protein binding, biliary excretion, metabolism, excretion of statins, renal elimination   Language of publication: English | Animal studies, case studies. |

+ cirrhosis is defined by clinical diagnosis, laboratory studies, imagining studies or liver biopsy

Research Question 2: In adult patients, how does liver cirrhosis change the safety, and cardiovascular efficacy of HMG-CoA reductase inhibitors?

| Inclusion criteria | Exclusion criteria |
| --- | --- |
| Study types: systematic reviews (only to review and extract relevant references), randomized controlled trials, cohort studies, case-controlled studies  Patient demographics: adult patients (>age 18), history of cirrhosis^+^ from any cause, any indications for statins, taking any statins  Outcome of interest:   - Efficacy outcomes: all-cause mortality, cardiovascular mortality, non-fatal myocardial infarction, non-fatal stroke, major cardiovascular adverse events, thrombotic events - Safety outcomes: myositis, rhabdomyolysis, deterioration of liver function tests, deterioration of existing liver condition, development of new liver conditions, gastrointestinal adverse effects, hemorrhagic stroke, diabetes mellitus, other statin-related adverse effects   Language of publication: English | Animal studies, case studies. |

+ cirrhosis is defined by clinical diagnosis, laboratory studies, imagining studies or liver biopsy

Research Question 3: In adult patients with liver cirrhosis taking HMG-CoA reductase inhibitors, what is the evidence of dose adjustment methods including Child-Pugh score, clinical gestalt, and MELD score for efficacy and safety outcomes?

| Inclusion Criteria | Exclusion Criteria |
| --- | --- |
| Study types: systematic reviews (only to review and extract relevant references), randomized controlled trials, cohort studies, case-controlled studies  Patient demographics: adult patients (>age 18), history of cirrhosis^+^ from any cause, any indications for statins, taking any statins  Interventions: Child-Pugh score, MELD score, clinical gestalt  Outcome of interest:   - Efficacy outcomes: all-cause mortality, cardiovascular mortality, non-fatal myocardial infarction, non-fatal stroke, major cardiovascular adverse events, thrombotic events, hospitalization rate due to any cause, complications of liver cirrhosis, mortality due to liver cirrhosis - Safety outcomes: myositis, rhabdomyolysis, deterioration of liver function tests, deterioration of existing liver condition, development of new liver conditions, gastrointestinal adverse effects, hemorrhagic stroke, diabetes mellitus, other statin-related adverse effects   Language of publication: English | Animal studies, case studies. |

+ cirrhosis is defined by clinical diagnosis, laboratory studies, imagining studies or liver biopsy

**Search Strategy:**

This systematic review intends to search available data to answer research questions 1,2 and 3. Same search strategy will be used to search for available evidence for all 3 questions. Articles will be screened using the inclusion/exclusion criteria for each individual question. Additional articles may be included in the review after the search is completed if they are considered relevant to the current study. The rationales for inclusion of these articles should be discussed in the manuscript.

- Search terms: statin* or atorvastatin* or lovastatin* or pravastatin* or rosuvastatin* or simvastatin* or fluvastatin* or lipitor or crestor or lescol or zocor or pravachol or mevacor or HMG-CoA reductase inhibitor* or pitavastatin* or livalo or hydroxymethylglutaryl-coa reductase inhibitor*
- Search terms: "Liver cirrhosis" or "cirrhosis" or "alcohol liver cirrhosis" or "biliary cirrhosis" or "compensated liver cirrhosis" or "decompensated liver cirrhosis" or "primary biliary cirrhosis"
- Excluded experimental cirrhosis, combination pills (ex: ezetimibe, ramipril), 7 statins searched: simvastatin, pravastatin, rosuvastatin, atorvastatin, fluvastatin, lovastatin, pitavastatin)
- Database: The following databases will be searched for available evidence: MEDLINE, EMBASE, Cochrane library (including Clinicaltrials.gov), CINAHL, SCOPUS
- The following databases will be searched for drug product monographs and unpublished pharmacokinetic studies specifically for research question 2: Health Canada Drug Product Database, Drugs@FDA.
- Review major statin trials and their inclusion/exclusion criteria (to check if liver cirrhosis was part of exclusion criteria)

**Study Selection Process:**

- Title/Abstract Screening: Using the above search strategy, articles identified will be screened by two reviewers independently. Reviewers will screen articles by titles and abstracts using the inclusion/exclusion criteria to assess if they are relevant to the research questions. If there are discrepancies in the decisions of which articles to be included or removed during this step, either the reviewer will meet and discuss until a unanimous decision is reached or a third reviewer will make the final decision.
- Full-Texts Screening: Two independent reviewers will screen the remaining articles by the full-text using the inclusion/exclusion criteria to select eligible studies to be considered in the systematic review. If there are discrepancies in their decisions in terms of which articles to be removed during the initial step, the reviewers will discuss until a unanimous decision is reached or a third reviewer will make the final decision.
- One reviewer will search Health Canada Drug Product Database and Drugs@FDA database for the seven statins stated above.
- PRISMA flow diagram will be used to present the process of main study selection.

**Data Extraction Process:**

Microsoft®Excel® spreadsheet will be used for data extraction.

The following data will be recorded: Authors/Publication year, study type, number of participants/duration of study, participant characteristics, inclusion/exclusion criteria, intervention, efficacy outcome, and safety outcome, and pharmacokinetics outcome.

**Study Assessment Process:**

Selected studies will be assessed for quality and bias independently by two reviewers. Randomized controlled trials will be assessed using Cochrane Collaboration tool for assessing risk of bias. Observational studies will be assessed using Newcastle-Ottawa Quality assessment scale. If there are discrepancies between the reviewers’ assessment, the reviewers will discuss until a unanimous decision is reached or a third reviewer will make the final decision.

**Data Analysis:**

If there is sufficient quantitative data collected from search, a meta-analysis will be performed for all research questions.

Qualitative data will be summarized and presented in a table if appropriate.

**References:**

1. Verbeeck RK. Pharmacokinetics and dosage adjustment in patients with hepatic dysfunction. European journal of clinical pharmacology. 2008 Dec 1;64(12):1147.
2. Food and Drug Administration. Guidance for industry. Pharmacokinetics in patients with impaired hepatic function: study design, data analysis, and impact on dosing and labeling. 2003. Available at: http://www.fda.gov/downloads/drugs/guidancecomplianceregulatoryinformation/guidances/ucm072123.pdf
3. Bosch J, Gracia-Sancho J, Abraldes JG. Cirrhosis as new indication for statins. Gut. 2020 May 1;69(5):953-62.
4. Wan S, Huang C, Zhu X. Systematic review with a meta-analysis: clinical effects of statins on the reduction of portal hypertension and variceal haemorrhage in cirrhotic patients. BMJ open. 2019 Jul 1;9(7):e030038.
5. Gu Y, Yang X, Liang H, Li D. Comprehensive evaluation of effects and safety of statin on the progression of liver cirrhosis: a systematic review and meta-analysis. BMC gastroenterology. 2019 Dec 1;19(1):231.
6. Ma X, Sun D, Li C, Ying J, Yan Y. Statin use and virus-related cirrhosis: a systemic review and meta-analysis. Clinics and Research in Hepatology and Gastroenterology. 2017 Oct 1;41(5):533-42.
7. Heart Protection Study Collaborative Group. MRC/BHF Heart Protection Study of cholesterol lowering with simvastatin in 20 536 high-risk individuals: a randomised placebo-controlled trial. The Lancet. 2002 Jul 6;360(9326):7-22.
8. LaRosa JC, Grundy SM, Waters DD, Shear C, Barter P, Fruchart JC, Gotto AM, Greten H, Kastelein JJ, Shepherd J, Wenger NK. Intensive lipid lowering with atorvastatin in patients with stable coronary disease. New England Journal of Medicine. 2005 Apr 7;352(14):1425-35.
9. Ridker PM, Danielson E, Fonseca FA, et al. Rosuvastatin to prevent vascular events in men and women with elevated C-reactive protein. New England Journal of Medicine. 2008;359(21):2195-2207. doi:10.1056/NEJMoa0807646
10. Schachter M. Chemical, pharmacokinetic and pharmacodynamic properties of statins: an update. Fundam Clin Pharmacol 2005 Feb;19(1):117-125.
